# Perioperative Use of Glucagon-Like Peptide-1 (GLP-1) Receptor Agonists Lower Readmission Rates After Coronary Artery Bypass Grafting (CABG) in Adults With Congenital Heart Disease

**DOI:** 10.7759/cureus.95767

**Published:** 2025-10-30

**Authors:** Jason H Yan, Joey McKinnerney, Taysir Al Janabi, Anwar Chahal, Adnan Malik, Michael N Vranian, Rahul Kashyap

**Affiliations:** 1 Cardiothoracic Surgery, Drexel University College of Medicine, Philadelphia, USA; 2 Department of Internal Medicine, Wellspan York Hospital, York, USA; 3 Department of Heart and Vascular, Wellspan York Hospital, York, USA; 4 Department of Research, Wellspan York Hospital, York, USA

**Keywords:** cabg, cad: coronary artery disease, congenital heart disease, glp-1, mortality

## Abstract

Background

Glucagon-like peptide-1 (GLP-1) receptor agonists (RA) are increasingly prescribed for type 2 diabetes and obesity, with emerging evidence of cardiovascular benefits. However, their impact on postoperative outcomes in adult congenital heart disease (CHD) patients undergoing coronary artery bypass grafting (CABG) remains underexplored.

Methods

We conducted a retrospective cohort study using de-identified patient data from the TriNetX Research Network from 2005 to 2025. Adult CHD patients (International Classification of Diseases, 10th Revision (ICD-10) codes Q20-Q28) who underwent CABG (Current Procedural Terminology (CPT) codes 1006208, 1006217, 1006200) were divided into two cohorts: those with documented perioperative GLP-1 RA use, defined as use within three months prior to or one month after CABG (Anatomical Therapeutic Chemical (ATC) code A10BJ), and those with no documented GLP-1 RA use (control cohort). Propensity score matching (1:1) was used to balance the cohorts based on race, ethnicity, sex, age at surgery, comorbidities (including diabetes, obesity, and heart failure), and CHD diagnoses.

Primary outcomes included one-year all-cause mortality and hospital readmission rates. Secondary outcomes included GLP-1-related complications (acute pancreatitis, gastroparesis, abnormal weight loss) and postoperative events (acute kidney injury (AKI), myocardial infarction (MI), transient ischemic attack (TIA), cerebral infarction, arrhythmia, and hypoglycemic events).

Results

A total of 720 patients were included after 1:1 propensity score matching; 360 patients remained in each group (GLP-1 RA users vs control). Baseline characteristics, including age, sex, and key comorbidities, were well balanced.

GLP-1 RA use was associated with a significantly lower one-year hospital readmission rate (odds ratio (OR) = 0.68; *p* = 0.0118), but no difference in one-year all-cause mortality (OR = 0.747; *p* = 0.3506). No significant differences were found in GLP-1 RA-related complications: gastroparesis (OR = 1.00; *p* = 1), abnormal weight loss (OR = 1.00; *p* = 1), or hypoglycemic events (OR = 1.103; *p* = 0.82). Postoperative outcomes, including AKI (OR = 0.97; *p* = 0.8612), MI (OR = 0.878; *p* = 0.4713), TIA (OR = 0.763; *p* = 0.5249), cerebral infarction (OR = 0.852; *p* = 0.5267), and arrhythmia (OR = 0.978; *p* = 0.8815), showed no significant differences between groups.

Conclusion

In adult CHD patients undergoing CABG, perioperative use of GLP-1 receptor agonists was associated with a significant reduction in one-year hospital readmission rates, without increased risk of mortality, postoperative complications, or known GLP-1-related adverse events.

## Introduction

Congenital heart disease (CHD) is a form of cardiovascular disease affecting over one million individuals worldwide [1]. Advances in interventional cardiac surgeries have significantly improved the repair and management of CHD, resulting in an adult population with a higher prevalence of CHD [2]. Additionally, CHD is significantly associated with developing type 2 diabetes mellitus (T2DM) after the age of 30 [3]. However, CHD’s long-term effect on cardiac outcomes in adult T2DM patients remains underexplored in the current literature.

The current standard of care for T2DM patients with underlying cardiovascular disease includes antihyperglycemic drugs metformin and sodium-glucose linked cotransporters 2 (SGLT-2) inhibitors. These therapies have demonstrated cardioprotective effects beyond their primary role for glycemic control [4,5]. Glucagon-like peptide-1 receptor agonists (GLP-1 RAs) are an emerging treatment for TD2M patients for weight loss that has shown improvements in cardiovascular outcome in those with existing cardiovascular disease (CVD) [6]. In multiple cardiovascular outcome trials, GLP-1 RAs showed a reduction of up to 14% in major adverse cardiovascular events (MACE) and 10% to 11% reduction in hospitalizations for heart failure [7]. Due to the increased prevalence of comorbidities in CHD and coronary artery bypass graft (CABG) patients, GLP-1 RAs may be a useful supplementary agent perioperatively in this high-risk group.

CABG surgeries are commonly performed, with over 200,000 of these surgeries performed in the United States annually. CABG is performed in the setting of atherosclerotic disease for patients with coronary artery disease (CAD). Despite its prevalence, it remains a high-risk procedure, with 14% of post-CABG patients presenting to the ED with postoperative complications [8]. CHD patients have increased risk factors for atherosclerotic cardiovascular disease (ASCVD), making this population group particularly relevant to study CABG outcomes [9]. GLP-1 RAs may improve CABG outcomes in high-risk CHD patients by offering a cardioprotective effect.

This study aimed to investigate the potential cardioprotective effects of perioperative GLP-1 RA use in adult patients with T2DM and a history of CHD undergoing CABG, investigating one-, three-, six-, and 12-month outcomes.

An abstract based on this study was accepted to the American Heart Association (AHA) Scientific Sessions 2025 for presentation.

## Materials and methods

Study design and data source

This study was a retrospective cohort analysis utilizing de-identified electronic health record data from the TriNetX Research Network. The data used in this study was collected on June 6, 2025, from the TriNetX Research Network, which provided access to electronic medical records (diagnoses, procedures, medications, laboratory values, genomic information) from approximately 154 million patients from 106 healthcare organizations. The study period spanned from 2005 to 2025. TriNetX, LLC was used as the data platform to conduct patient queries, extract relevant cohorts, and perform statistical comparisons across groups.

This retrospective study is exempt from informed consent. The data reviewed is a secondary analysis of existing data, does not involve intervention or interaction with human subjects, and is de-identified per the de-identification standard defined in Section §164.514(a) of the Health Insurance Portability and Accountability Act (HIPAA) Privacy Rule. The process by which the data is de-identified is attested to through formal determination by a qualified expert as defined in Section §164.514(b)(1) of the HIPAA Privacy Rule.

Study objectives

Primary Objective

The primary objective was to determine whether perioperative GLP-1 RA use is associated with differences in (1) all-cause mortality and (2) hospital readmission rates at one, three, six, and 12 months following CABG.

Secondary Objectives

The secondary objectives were: (1) to assess the incidence of GLP-1 RA-related adverse events, including acute pancreatitis, gastroparesis, abnormal weight loss, and hypoglycemia; (2) to compare postoperative complications between GLP-1 RA users and non-users, including acute kidney injury, myocardial infarction, transient ischemic attack, cerebral infarction, and arrhythmia.

Study population, variables, and outcomes

The study included adult patients (aged 18 years or older) diagnosed with CHD, identified using International Classification of Diseases, 10th Revision (ICD-10) codes Q20-Q28, who underwent CABG, identified by Current Procedural Terminology (CPT) codes 1006208, 1006217, and 1006200. Two groups were constructed: patients who had documented use of GLP-1 RA (Anatomical Therapeutic Chemical (ATC) code A10BJ) within three months prior to or one month after CABG - defined as perioperative use (exposure group) - and those with no documented GLP-1 RA use (control group). To reduce confounding, propensity score matching was conducted in a 1:1 ratio using a greedy nearest-neighbor algorithm without replacement. Matching was based on demographic variables (age at surgery, sex, race, and ethnicity) and clinical covariates, including comorbid diabetes, BMI, and CHD subtype diagnoses (Table 1). CHD subtype classifications were based on ICD-10-CM codes recorded as present up to one day before the index CABG and included congenital malformations of cardiac chambers and connections (Q20), cardiac septa (Q21), pulmonary and tricuspid valves (Q22), aortic and mitral valves (Q23), other congenital malformations of the heart (Q24), great arteries (Q25), great veins (Q26), peripheral vascular system (Q27), and other congenital malformations of the circulatory system (Q28). Post-matching balance was assessed through comparison of baseline characteristics between the cohorts (Table 2).

**Table 1:** Demographic Variables Included in Propensity Score Matching ICD-10-CM: International Classification of Diseases, 10th Revision, Clinical Modification

Variable	Definition
Age	N/A
Sex	N/A
Ethnicity	N/A
Race	N/A
Diabetes mellitus	ICD-10-CM: E08–E13
Hypertensive diseases	ICD-10-CM: I10–I1A
Acute myocardial infarction	ICD-10-CM: I21
Chronic ischemic heart disease	ICD-10-CM: I25
Cerebrovascular diseases	ICD-10-CM: I60–I69
Heart failure	ICD-10-CM: I50
Nonrheumatic aortic valve disorders	ICD-10-CM: I35
Angina pectoris	ICD-10-CM: I20
Overweight, obesity and other hyperalimentation	ICD-10-CM: E65–E68
Congenital heart and vascular malformations	ICD-10-CM: Q20–Q28
Peripheral vasodilators	VA: CV500
Angiotensin II inhibitors	VA: CV805
Beta blockers	VA: CV100
Antiarrhythmics	VA: CV300
Diuretics	VA: CV700
Calcium channel blockers	VA: CV200
BMI 19.9 or less	ICD-10-CM: Z68.1
BMI 20–29	ICD-10-CM: Z68.2
BMI 30–39	ICD-10-CM: Z68.3
BMI ≥40	ICD-10-CM: Z68.4

**Table 2:** Baseline demographic and clinical characteristics of adult CHD patients undergoing CABG, comparing GLP-1 RA users and non-users after propensity score matching. GLP-1 RA: glucagon-like peptide-1 receptor agonist, CHD: congenital heart disease, CABG: coronary artery bypass grafting

	GLP-1 RA user: Patient Count (%) (N=360)	Non-GLP-1 RA user: Patient Count (%) (N=360)	After: p-Value
Demographic			
Age at Index, mean (± standard deviation)	62.78 (9.38)	62.4 ± 9.79	0.63
Male	253 (70.28%)	256 (71.11%)	0.81
Female	93 (25.83%)	90 (25%)	0.80
Unknown Gender	14 (3.89%)	14 (3.89%)	1.00
Race			
White	285 (79.17%)	289 (80.28%)	0.71
Black or African American	23 (6.39%)	21 (5.83%)	0.76
Asian	11 (3.06%)	≤ 10 (2.78%)	0.82
Native Hawaiian or Other Pacific Islander	≤ 10 (2.78%)	≤ 10 (2.78%)	1.00
American Indian or Alaska Native	0 (0%)	0 (0%)	1.00
Other Race	≤ 10 (2.78%)	≤ 10 (2.78%)	1.00
Unknown Race	28 (7.78%)	28 (7.78%)	1.00
Ethnicity			
Hispanic or Latino	28 (7.78%)	26 (7.22%)	0.78
Not Hispanic or Latino	268 (74.44%)	278 (77.22%)	0.38
Unknown Ethnicity	64 (17.78%)	56 (15.56%)	0.42
Comorbidity			
Chronic ischemic heart disease	356 (98.89%)	354 (98.33%)	0.52
Diabetes mellitus	335 (93.06%)	335 (93.06%)	1.00
Hypertensive diseases	330 (91.67%)	335 (93.06%)	0.48
Overweight and obesity	232 (64.44%)	235 (65.28%)	0.81
Heart failure	164 (45.56%)	183 (50.83%)	0.16
Cerebrovascular diseases	153 (42.50%)	170 (47.22%)	0.20
Nonrheumatic aortic valve disorders	152 (42.22%)	148 (41.11%)	0.76
Acute myocardial infarction	147 (40.83%)	149 (41.39%)	0.88
Angina pectoris	136 (37.78%)	134 (37.22%)	0.88
BMI			
Body mass index [BMI] 19.9 or less, adult	≤ 10 (2.78%)	≤ 10 (2.78%)	1.00
Body mass index [BMI] 20-29, adult	40 (11.11%)	47 (13.06%)	0.42
Body mass index [BMI] 30-39, adult	132 (36.67%)	149 (41.39%)	0.19
Body mass index [BMI] 40 or greater, adult	47 (13.06%)	52 (14.44%)	0.59
CHD Diagnosis			
Congenital malformations of cardiac septa	70 (19.44%)	69 (19.17%)	0.92
Congenital malformations of aortic and mitral valves	68 (18.89%)	64 (17.78%)	0.70
Congenital malformations of great arteries	34 (9.44%)	40 (11.11%)	0.46
Congenital malformations of cardiac chambers and connections	≤ 10 (2.78%)	≤ 10 (2.78%)	1.00
Congenital malformations of pulmonary and tricuspid valves	≤ 10 (2.78%)	≤ 10 (2.78%)	1.00
Congenital malformations of great veins	≤ 10 (2.78%)	0 (0%)	>0.01
Other congenital malformations of heart	67 (18.61%)	61 (16.94%)	0.55
Other congenital malformations of peripheral vascular system	14 (3.89%)	18 (5%)	0.47
Other congenital malformations of circulatory system	10 (2.78%)	10 (2.78%)	1.00
Medication			
Beta blockers/related	333 (92.50%)	332 (92.22%)	0.89
Antiarrhythmics	290 (80.56%)	287 (79.72%)	0.78
Diuretics	273 (75.83%)	266 (73.89%)	0.55
Calcium channel blockers	252 (70%)	248 (68.89%)	0.75
Angiotensin II inhibitors	165 (45.83%)	183 (50.83%)	0.18
Peripheral vasodilators	61 (16.94%)	54 (15%)	0.48

The primary outcomes assessed were all-cause mortality and hospital readmission, evaluated at one-, three-, six-, and 12-month time points following CABG. Secondary outcomes (ICD-10 codes in Table 3) included known adverse events associated with GLP-1 RAs and common postoperative complications, including acute pancreatitis, gastroparesis, abnormal weight loss, acute kidney injury, myocardial infarction, transient ischemic attack, cerebral infarction, and hypoglycemic events. Each outcome was also evaluated at one, three, six, and 12 months postoperatively to assess the time-dependent impact of GLP-1 RA use.

**Table 3:** ICD-10 diagnosis codes used to define study outcomes, including GLP-1-related and post-operative complications. GLP-1: glucagon-like peptide-1, ICD-10: International Classification of Diseases, 10th Revision

Outcome / Complication	ICD-10 Code(s)
Acute Pancreatitis	K85
Gastroparesis	K31.84
Abnormal Weight Loss	R63.4
Acute Kidney Injury (AKI)	N17
Myocardial Infarction (MI)	I21
Transient Ischemic Attack (TIA)	G45.9
Cerebral Infarction	I63
Hypoglycemic Event	E16.1, E16.2

Statistical analysis

Descriptive statistics were used to summarize demographic and clinical characteristics of the study cohorts before and after propensity score matching. Statistical comparisons between matched cohorts were performed using built-in analytic functions within the TriNetX platform, which uses validated algorithms and ensures compliance with HIPAA and institutional data governance standards. For each outcome, absolute event rates were calculated, and the risk difference (RD), risk ratio (RR), and odds ratio (OR) with corresponding 95% confidence intervals (CIs) were reported to quantify associations between GLP-1 RA use and clinical outcomes. Logistic regression models were used within the TriNetX environment to generate odds ratios and assess statistical significance. Due to the small patient population size and limitations within TriNetX designed to protect the confidentiality of healthcare organizations (HCOs), any patient count in the outcome table less than or equal to 10 was reported as "≤10" and analyzed as "10." A two-sided p-value of less than 0.05 was considered statistically significant.

## Results

Demographics and baseline characteristics

The study consisted of two groups: CHD patients undergoing CABG with GLP-1 RA therapy versus CHD patients undergoing CABG patients without GLP-1 RA therapy. We identified 364 patients for the GLP-1 RA cohort, and 36,634 patients without GLP-1 RA before propensity score (Table 3) matching. After matching, there were 360 patients in each group.

The demographic and baseline characteristics of the two groups including age, gender identity, race, ethnicity, comorbidities, and medications are presented in Table 1. After propensity score matching, the demographic and baseline characteristics were well-matched with no significant differences between the two groups. The average age was approximately 62 years old, and many of them had a history of chronic ischemic heart disease, T2DM, or hypertensive disease.

One-month outcomes

The two primary outcomes measured were 30-day hospital readmission rates and 30-day all-cause mortality (Table 4). The hospital readmission rates showed a statistically significant lower rate in the GLP-1 RA group. There was no significant difference between the two groups for 30-day mortality. Secondary outcomes did not show a meaningful difference between the two groups for stroke, transient ischemic attack, myocardial infarction, and arrhythmia. The GLP-1 RA group was not associated with increased risk of known adverse side effects related to GLP-1 RA use including gastroparesis, abnormal weight loss, acute kidney injury, and hypoglycemic events.

**Table 4:** Postoperative outcomes at 30 days, three months, six months, and 12 months in GLP-1 RA users versus non-users following CABG, with corresponding risk differences, risk ratios, and odds ratios. GLP-1 RA: glucagon-like peptide-1 receptor agonist, CABG: coronary artery bypass grafting, TIA: transient ischemic attack

	GLP-1 RA user: Patient Count (%)(N=360)	Non-GLP-1 RA user: Patient Count (%) (N=360)	Risk Difference	95 % CI	p-value	Odds Ratio	95 % CI
30-days							
Stroke	17 (4.72%)	21 (5.83%)	-1.11%	(-4.38%,2.15%)	0.51	0.8	(0.42,1.54)
TIA	≤ 10 (2.78%)	≤ 10 (2.78%)	0%	(-2.4%,2.4%)	1.0	1.0	(0.41,2.43)
Gastroparesis	≤ 10 (2.78%)	≤ 10 (2.78%)	0%	(-2.41%,2.4%)	1.0	1.0	(0.41,2.43)
Mortality	≤ 10 (2.78%)	14 (3.89%)	-1.11%	(-3.73%,1.51%)	0.41	0.71	(0.31,1.61)
Abnormal weight loss	≤ 10 (2.78%)	10 (2.78%)	0%	(-2.41%,2.4%)	1.0	1.0	(0.41,2.43)
Myocardial infarction	54 (15.00%)	58 (16.11%)	-1.11%	(-6.41%,4.18%)	0.68	0.92	(0.61,1.38)
Hospital readmission	146 (40.56%)	179 (49.72%)	-9.17%	(-16.41%, -1.93%)	0.01	0.69	(0.51,0.93)
Arrhythmia	140 (38.89%)	146 (40.56%)	-1.67%	(-8.81%,5.48%)	0.65	0.93	(0.69,1.26)
Acute pancreatitis	≤ 10 (2.78%)	≤ 10 (2.78%)	0%	(-2.4%,2.4%)	1.0	1.0	(0.41,2.43)
Hypoglycemic event	≤ 10 (2.78%)	≤ 10 (2.78%)	0%	(-2.4%,2.4%)	1.0	1.0	(0.41,2.43)
Acute kidney injury	56 (15.56%)	58 (16.11%)	-0.56%	(-5.888%,4.777%)	0.84	0.96	(0.64,1.43)
3-months							
Stroke	24 (6.67%)	28 (7.78%)	-1.11%	(-4.89%,2.67%)	0.56	0.85	(0.48,1.49)
TIA	≤ 10 (2.78%)	≤ 10 (2.78%)	0%	(-2.4%,2.4%)	1.0	1.0	(0.41,2.43)
Gastroparesis	≤ 10 (2.78%)	≤ 10 (2.78%)	0%	(-2.4%,2.4%)	1.0	1.0	(0.41,2.43)
Mortality	12 (3.33%)	16 (4.44%)	-1.11%	(-3.93%,1.71%)	0.44	0.74	(0.35,1.59)
Abnormal weight loss	≤ 10 (2.78%)	≤ 10 (2.78%)	0%	(-2.4%,2.4%)	1.0	1.0	(0.41,2.43)
Myocardial infarction	61 (16.94%)	70 (19.44%)	-2.50%	(-8.13%,3.13%)	0.38	0.85	(0.58,1.24)
Hospital readmission	168 (46.67%)	199 (55.28%)	-8.61%	(-15.89%,-1.34%)	0.02	0.71	(0.53,0.95)
Arrhythmia	161 (44.72%)	163 (45.28%)	-0.56%	(-7.82%,6.71%)	0.88	0.98	(0.73,1.31)
Acute pancreatitis	≤ 10 (2.78%)	≤ 10 (2.78%)	0%	(-2.4%,2.4%)	1.0	1.0	(0.41,2.43)
Hypoglycemic event	≤ 10 (2.78%)	≤ 10 (2.78%)	0%	(-2.4%,2.4%)	1.0	1.0	(0.41,2.43)
Acute kidney injury	69 (19.17%)	69 (19.17%)	0%	(-5.75%,5.75%)	1.0	1.0	(0.69,1.45)
6-months							
Stroke	29 (8.06%)	34 (9.44%)	-1.39%	(-5.52%,2.74%)	0.51	0.84	(0.5,1.41)
TIA	≤ 10 (2.78%)	12 (3.33%)	-0.56%	(-3.07%,1.96%)	0.67	0.83	(0.35,1.94)
Gastroparesis	≤ 10 (2.78%)	≤ 10 (2.78%)	0%	(-2.4%,2.4%)	1.0	1.0	(0.41,2.43)
Mortality	15 (4.17%)	19 (5.28%)	-1.11%	(-4.21%,1.99%)	0.48	0.78	(0.39,1.56)
Abnormal weight loss	≤ 10 (2.78%)	≤ 10 (2.78%)	0%	(-2.4%,2.4%)	1.0	1.0	(0.41,2.43)
Myocardial infarction	67 (18.61%)	76 (21.11%)	-2.50%	(-8.33%,3.33%)	0.40	0.85	(0.59,1.23)
Hospital readmission	187 (51.94%)	221 (61.39%)	-9.44%	(-16.65%,-2.24%)	0.01	0.68	(0.51,0.91)
Arrhythmia	168 (46.67%)	169 (46.94%)	-0.28%	(-7.57%,7.01%)	0.94	0.99	(0.74,1.33)
Acute pancreatitis	≤ 10 (2.78%)	≤ 10 (2.78%)	0%	(-2.4%,2.4%)	1.0	1.0	(0.41,2.43)
Hypoglycemic event	≤ 10 (2.78%)	≤ 10 (2.78%)	0%	(-2.4%,2.4%)	1.0	1.0	(0.41,2.43)
Acute kidney injury	81 (22.50%)	81 (22.50%)	0%	(-6.1%,6.1%)	1.0	1.0	(0.71,1.42)
1 year							
Stroke	32 (8.89%)	37 (10.28%)	-1.39%	(-5.69%,2.91%)	0.53	0.85	(0.52,1.4)
TIA	10 (2.78%)	13 (3.61%)	-0.83%	(-3.4%,1.74%)	0.52	0.76	(0.33,1.76)
Gastroparesis	≤ 10 (2.78%)	≤ 10 (2.78%)	0%	(-2.4%,2.4%)	1.0	1.0	(0.41,2.43)
Mortality	19 (5.28%)	25 (6.94%)	-1.67%	(-5.16%,1.83%)	0.35	0.75	(0.4,1.38)
Abnormal weight loss	≤ 10 (2.78%)	≤ 10 (2.78%)	0%	(-2.4%,2.4%)	1.0	1.0	(0.41,2.43)
Myocardial infarction	75 (20.83%)	83 (23.06%)	-2.22%	(-8.27%,3.82%)	0.47	0.88	(0.62,1.25)
Hospital readmission	202 (56.11%)	235 (65.28%)	-9.17%	(-16.27%,-2.06%)	0.01	0.68	(0.5,0.92)
Arrhythmia	177 (49.17%)	179 (49.72%)	-0.56%	(-7.86%,6.75%)	0.88	0.98	(0.73,1.31)
Acute pancreatitis	≤ 10 (2.78%)	≤ 10 (2.78%)	0%	(-2.4%,2.4%)	1.0	1.0	(0.41,2.43)
Hypoglycemic event	11 (3.06%)	≤ 10 (2.78%)	0.28%	(-2.18%,2.74%)	0.82	1.10	(0.46,2.63)
Acute kidney injury	85 (23.61%)	87 (24.17%)	-0.56%	(-6.79%,5.67%)	0.86	0.97	(0.69,1.37)

Three-month outcomes

For three-month outcomes, the primary outcome of readmission rates remained significantly lower in the GLP-1 RA group. Mortality at three months did not change between the two groups. Among secondary outcomes, no meaningful difference was observed for stroke, transient ischemic attack, myocardial infarction, and arrhythmia. Additionally, the GLP-1 RA group did not have increased risks of gastroparesis, abnormal weight loss, acute kidney injury, or hypoglycemic episodes.

Six-month outcomes

At six months, the GLP-1 RA group continued to show a statistically significant reduction in readmissions, while mortality remained similar between the groups. The secondary endpoints: stroke, transient ischemic attack, myocardial infarction, and arrhythmia continued to show no significant variation. Adverse effects between the groups remained statistically nonsignificant, such as gastroparesis, abnormal weight loss, acute kidney injury, and hypoglycemia.

One-year outcomes

At one year, the trend continued of significantly lower readmissions in the GLP-1 RA cohort, while mortality rates still showed no significant difference. No significant difference was detected in secondary outcomes: stroke, transient ischemic attack, myocardial infarction, and arrhythmia. As noted at earlier intervals, the GLP-1 RA cohort did not experience increased risks of GLP-1 RA-associated adverse effects. Gastroparesis, abnormal weight loss, acute kidney injury, or hypoglycemia showed no significant difference.

## Discussion

In this retrospective matched cohort study, CABG GLP-1 RA therapy is associated with a decrease in hospital readmission rates throughout the entire one-year postoperative period. The analysis also highlights the safety of GLP-1 RAs, showing no increased frequency of adverse events associated with GLP-1 RA therapy including acute pancreatitis, delayed gastric emptying and hypoglycemia. Additionally, there is no statistically significant difference in association with one-, three-, six-, and 12-month all-cause mortality rates between GLP-1 RA users and non-users. Our study findings are in concordance with existing research, suggesting an association with perioperative GLP-1 RA usage and decreased hospital readmission rates for diabetic patients who underwent surgical procedures [10,11]. GLP-1 RA’s cardioprotective effect was also demonstrated in a recent meta-analysis that GLP-1 RA significantly reduces mortality and hospitalization in patients with heart failure with preserved ejection fraction (HFpEF) [12]. The mechanism by which GLP-1 RAs improves surgical outcomes is likely through their effects on glycemic control, inflammation reduction, and weight management [13,14].

The data in our study showed GLP-1 RA is not associated with increased risk of known adverse effects such as AKI, acute pancreatitis and hypoglycemic event compared to the control group. While not found in our study, it was shown previously that AKI can occur due to nausea or vomiting-induced dehydration in some rare cases [15]. Previous literature supports the safety of GLP-1 RA therapy in cardiac procedures during the perioperative period when patients receive proper management, as GLP-1 RAs showed significantly lower postoperative blood glucose levels, similar or lower levels of heart rate arrhythmias, critically low blood sugar events, and decreased need for serious interventions compared to those receiving insulin alone [16]. The administration of intravenous GLP-1 analogs during surgery has shown no increased risk of gastrointestinal or respiratory complications according to recent clinical trials even though concerns about delayed gastric emptying and aspiration risk have been raised for liraglutide and semaglutide [17]. This is consistent with our data that GLP-1 RA is likely safe for adult CHD patients undergoing invasive CABG.

In a previous study, it was shown that GLP-1 RAs have a cardioprotective effect in ST-elevation myocardial infarction (STEMI) patients with reduced all-cause mortality [18]. However, our finding of no association in one-, three-, six-, and 12-month mortality between GLP-1 RA users and non-users post-surgery suggests that mortality benefits may not be the same for adult CHD patients undergoing critical cardiac procedures such as CABG. In adult congenital heart disease patients undergoing CABG, mortality is often influenced by anatomical and procedural complexities that may overshadow short-term pharmacologic effects [19]. Therefore, the absence of mortality benefit should be interpreted in the context of these factors and does not negate the potential long-term advantages of GLP-1 RA therapy, consistent with the ongoing morbidity and mortality experienced by adult CHD patients many years after surgical correction of their congenital heart disease [20].

**Figure 1:**
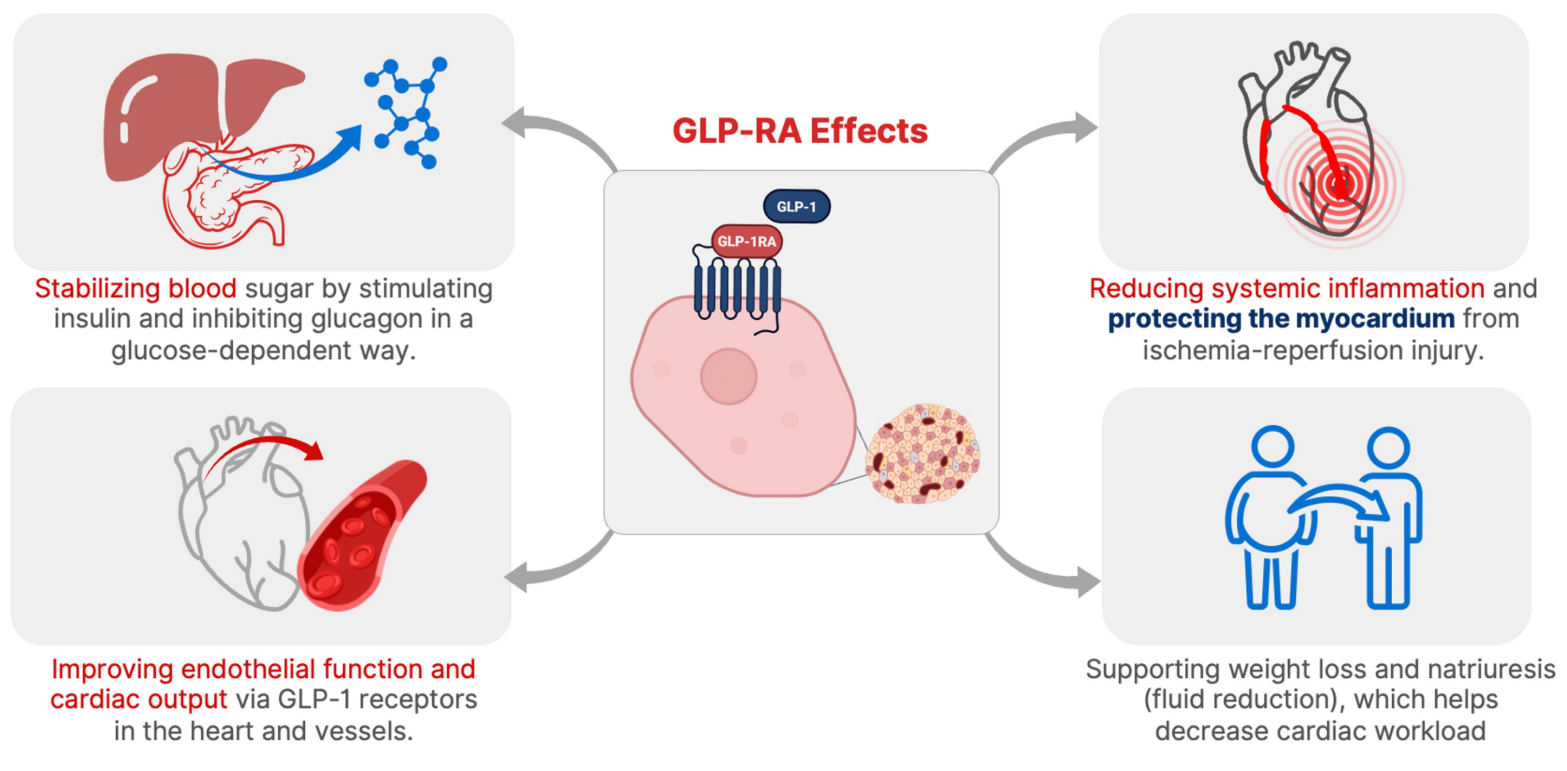
Potential Mechanisms Linking GLP-1 Receptor Agonists to Reduced Hospital Readmission Image Credits: Jason Yan (adapted from [21-23]) GLP-1: Glucagon-like peptide-1

The observed association reduction in hospital readmission among GLP-1 RA users may be attributable to the multifaceted cardiometabolic benefits of GLP-1 receptor agonists (Figure 1). These agents exert antihyperglycemic effects through glucose-dependent insulin secretion and inhibition of glucagon release, which collectively improve perioperative glycemic stability, a known factor associated with better surgical outcomes and reduced complications [21]. Additionally, GLP-1 RAs possess anti-inflammatory and endothelial-stabilizing properties that may attenuate postoperative systemic inflammation and promote myocardial protection [22], potentially reducing the incidence of complications that necessitate rehospitalization. GLP-1 receptors are also expressed in cardiac and vascular tissues, and activation has been shown to improve myocardial function, reduce ischemia-reperfusion injury, and enhance cardiac output [23]. Furthermore, by supporting weight loss and reducing fluid retention through natriuretic effects, GLP-1 RAs may reduce the burden on the myocardium, particularly in patients with underlying structural abnormalities common in adult congenital heart disease.

Strengths and limitations

This study has several strengths. We investigated adults with congenital heart disease who undergo complex cardiac surgery which is not well-studied in prior cardiovascular studies [24]. Our analysis benefits from a large nationally representative real-world dataset which enables better generalization across different healthcare settings and patient demographics. Our study included a relatively large patient population size considering that our study on CHD patients undergoing CABG with the use of GLP-1 agonist is rare. By using a large database like TriNetX, we are able to leverage the large number of patient population even for a niche population. Standardized electronic health records and validated coding algorithms improved the reliability and reproducibility of our data. Moreover, propensity score matching was employed to reduce confounding by indication, improving the comparability between GLP-1 RA users and non-users by accounting for multiple baseline variables. After matching, GLP-1 RA users and non-users usually show well-balanced covariates, validated by p-values. 

However, since this is a retrospective observational cohort analysis, we cannot establish causality, and the findings should be interpreted as a statistically significant association and not causal. Another limitation was that our exposure definition relied on prescription records and diagnosis codes without access to pharmacy fill data or direct confirmation of medication adherence. As a result, misclassification bias may exist due to uncertainty regarding whether patients complied with the prescribed GLP-1 RAs intake and the duration of therapy.

Additionally, while our study included up to 12 months of follow-up, this period may be insufficient to capture the full spectrum of long-term postoperative complications and mortality, particularly in patients with complex congenital and acquired cardiovascular disease. The relatively small number of GLP-1 RA users with congenital heart diseases within our cohort further limits statistical power for detecting differences in less common adverse events or subgroup effects. TriNetX reports any patient counts ≤10 as "≤10" to protect patient privacy as part of the de-identification process. While this safeguards confidentiality, it may obscure the exact frequency of rare outcomes and limit the precision of statistical estimates or subgroup analyses, reducing the ability to detect small but clinically meaningful differences between groups. Sample representativeness may be limited because the study population includes only patients from healthcare organizations participating in TriNetX, which could affect the generalizability of findings to other clinical settings. We also acknowledge the possibility of indication bias, as patients prescribed GLP-1 RAs may have been under closer metabolic surveillance or received more aggressive cardiometabolic management compared to non-users. Also, cause-specific mortality data were not available. Distinguishing cardiovascular from non-cardiovascular deaths could have provided more targeted insight into the potential protective mechanisms of GLP-1 RA therapy in the perioperative setting. Nevertheless, these findings provide an important foundation for future prospective studies to investigate the role of GLP-1 RAs in improving surgical outcomes among high-risk cardiovascular populations.

This study utilized built-in analytic functions within the TriNetX platform for statistical comparisons and modeling. A potential limitation of this approach is that the authors did not have the ability to customize or modify the underlying algorithms, which may impact flexibility in analysis and the exploration of alternative statistical methods.

Future research should prioritize prospective randomized controlled trials to validate the observed reduction in readmissions and the long-term impact of GLP-1 RAs on survival and quality of life in adult CHD surgical patients. Ideally, longer-term postoperative follow-up beyond 12 months is necessary to assess sustained benefits or risks associated with GLP-1 RA therapy in this unique patient population. Additionally, investigations into optimal perioperative timing, formulations [intravenous versus subcutaneous], and patient selection criteria will be critical to maximize therapeutic benefit while minimizing risk.

## Conclusions

Our study highlights the association between GLP-1 receptor agonist use and reduced hospital readmissions in adult patients with congenital heart disease undergoing coronary artery bypass grafting, suggesting a potential benefit in perioperative management. The observed associations may reflect the cardiometabolic, anti-inflammatory, and weight-modulating effects of GLP-1 RAs, which could contribute to improved early postoperative outcomes. No statistically significant differences in mortality were observed in this population, indicating that mortality benefits may be limited or influenced by other clinical factors. Overall, these findings support the safety of GLP-1 RA use in this high-risk surgical cohort and provide a basis for future prospective studies to further evaluate their potential role in optimizing postoperative recovery and long-term care.
